# From Silicosis to Systemic Sclerosis: Erasmus Syndrome in a Young Galamseyer—Case Report

**DOI:** 10.1155/crdm/4538413

**Published:** 2026-04-29

**Authors:** Solomon Gyabaah, Seth Kyei-Fram, Kojo Awotwi Hutton Mensah, Osei Yaw Asamoah, Clement Ohene Owusu, Divine Aseye Yao Amenuke, Yaw Adu-Boakye, Martin Kofi Agyei

**Affiliations:** ^1^ Department of Medicine, Komfo Anokye Teaching Hospital, Ashanti Region, Kumasi, Ghana, kathhsp.org; ^2^ Department of Medicine, Kwame Nkrumah University of Science and Technology, Ashanti Region, Kumasi, Ghana, knust.edu.gh

**Keywords:** case report, Erasmus syndrome, silicosis, systemic sclerosis

## Abstract

Erasmus syndrome is an uncommon condition marked by the onset of systemic sclerosis (SSc) due to extended exposure to silica. In this case, we highlight the significant health risks associated with silica exposure in artisanal mining (galamsey), leading to the development of Erasmus syndrome. We present the case of a 33‐year‐old male artisanal miner with a 14‐year work history who presented with a 2‐year history of recurrent cough, difficulty breathing, and fever. He later developed hypopigmentation and skin thickening on his face, neck, torso, and knees, along with a productive cough and low‐grade fever. He visited multiple healthcare facilities on account of the above complaints. At one of such visits, he was prescribed antituberculosis (TB) medication even though the sputum for GeneXpert was negative. On examination, he was in respiratory distress with an oxygen saturation of 78% on room air and required oxygen via a nonrebreather mask at 10 L/min, which raised the oxygen saturation to 94%, and had bronchial breath sounds in the right lung zones. Vocal resonance was increased bilaterally in the middle lung zones. He had salt‐and‐pepper dermopathy with tight and adherent skin on his face, chest, back, and knees. He additionally had microstomia, rat bite sign of the fingers, and Raynaud’s phenomenon. Antinuclear antibody by indirect immunofluorescence on hep‐2 cells was positive (1:320), and Antitopoisomerase 1 antibodies were also positive. Chest CT findings were consistent with complicated silicosis. The patient was diagnosed with Erasmus syndrome and started on immunosuppressive therapy. We highlight the significant health risks associated with silica exposure in artisanal mining (galamsey), leading to the development of Erasmus syndrome. Preventing silica exposure remains crucial, necessitating stringent workplace safety measures and occupational health practices to protect individuals in high‐risk professions.

## 1. Introduction

Erasmus syndrome is an uncommon condition marked by the onset of systemic sclerosis (SSc) due to extended exposure to silica. Dr L. D. Erasmus initially reported SSc cases in South African gold miners [1]. The hypothesis underlying the development of SSc suggests that silica exposure may stimulate the innate immune response in the body, resulting in lung inflammation, activation of adaptive immune response, and generation of self‐reactive T‐lymphocytes that exhibit resistance to apoptosis [2, 3].

Prolonged exposure to silica, commonly seen in miners and stone cutters, triggers an inflammatory response that causes irreversible interstitial lung disease [4]. Professionals in various fields, such as dental technicians, and individuals working with quartz and abrasive powder also experience Erasmus syndrome [2]. SSc is an autoimmune disease with multiple organ involvement characterized by skin tightening, musculoskeletal pain, exertional dyspnea, weight loss, and neuropathy [5]. Galamsey is a recognized term for artisanal mining in Ghana. The word is derived from the phrase “gather them and sell.” We report the case of a young “galamseyer” with Erasmus Syndrome.

## 2. Case Presentation

This case involves a 33‐year‐old male artisanal miner with a 14‐year work history who presented with an occasional nonproductive cough of 5 years duration. However, the cough became persistent and productive of whitish sputum associated with recurrent breathlessness and low‐grade fever over the 2‐year period prior to presentation. The patient acknowledged not using personal protective equipment while working. He had no prior comorbidities and initially experienced severe bilateral knee joint pain, which was worse in the mornings. He later developed hypopigmentation and skin thickening on his face, neck, torso, and knees. He visited multiple healthcare facilities on account of the above complaints. At one of such visits, he was prescribed antituberculosis (TB) medication even though the sputum for GeneXpert was negative. His symptoms of exertional dyspnea and cough progressed despite completing a 6‐month course of anti‐TB medications. This prompted the last attending physician to refer the patient to the Komfo Anokye Teaching Hospital.

On examination, he was in respiratory distress with an oxygen saturation of 78% on room air and required oxygen via a nonrebreather mask at 10 L/min, which raised the oxygen saturation to 94%. He was moderately pale, had reduced breath sound intensity (air entry) in the upper and middle lung zones, and had bronchial breath sounds in the right lung zones. Vocal resonance was increased bilaterally in the middle lung zones. He had salt‐and‐pepper dermopathy with tight and adherent skin on his face, chest, back, and knees (Figures 1(a), 1(b), and 1(c)). He additionally had microstomia, rat bite sign of the fingers, and Raynaud’s phenomenon. The Raynaud’s phenomenon had been present for close to a year. However, the patient did not have sclerodactyly. He was conscious and alert.

FIGURE 1(a–c) Tightened skin with leukoderma, consistent with a “salt‐and‐pepper” appearance.

**Figure:**
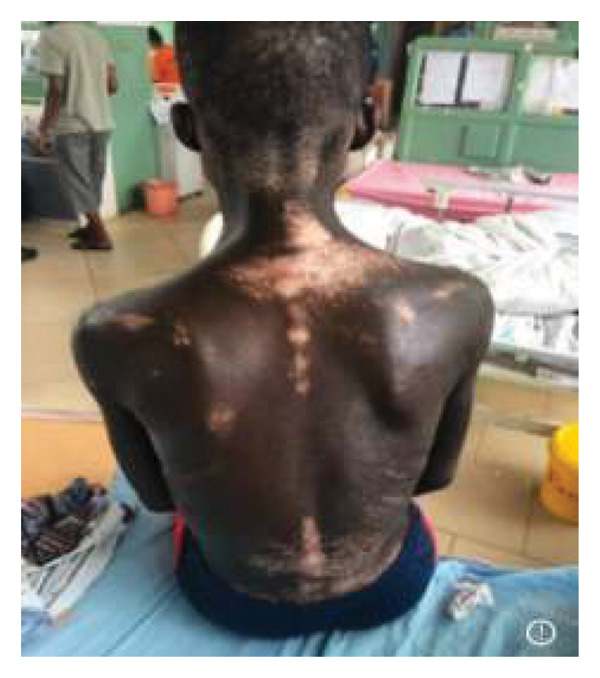
(a)

**Figure:**
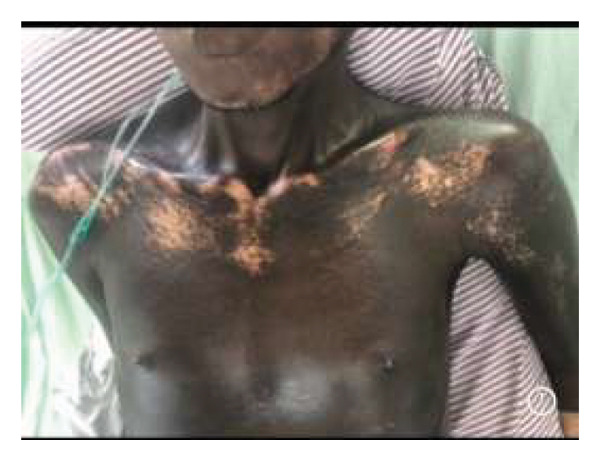
(b)

**Figure:**
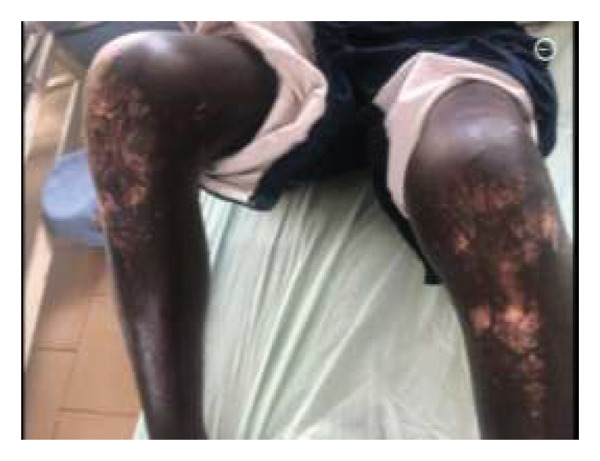
(c)

Laboratory investigations showed a hemoglobin level of 11.5 g/dL, an erythrocyte sedimentation rate of 67 mm fall in the first hour, and a C‐reactive protein of 41.9 mg/dL. Liver and renal function tests were normal, and negative results were obtained for HIV, hepatitis B, and hepatitis C serology. Pulmonary TB was considered as part of the differential diagnosis; however, the sputum for GeneXpert was negative. Antinuclear antibody by indirect immunofluorescence on hep‐2 cells was positive (1:320), and Antitopoisomerase 1 antibodies were also positive.

Chest CT scan showed mass‐like consolidations with spiculated margins involving both upper lobes and the superior segments of the lower lobes, with parenchymal calcifications seen within the areas of consolidation. There was associated traction bronchiectasis, fibrotic changes, and subpleural cystic changes with mediastinal and left perihilar lymph nodes seen with eggshell‐type calcification, findings consistent with complicated silicosis (Figures 2(a) and 2(b)).

FIGURE 2(a) Fibrosis of the lung with tram‐tract appearance and signet ring appearance of the lung, and reduced lung volume on the left. (b) Presence of radio‐opaque silica in both lungs.

**Figure:**
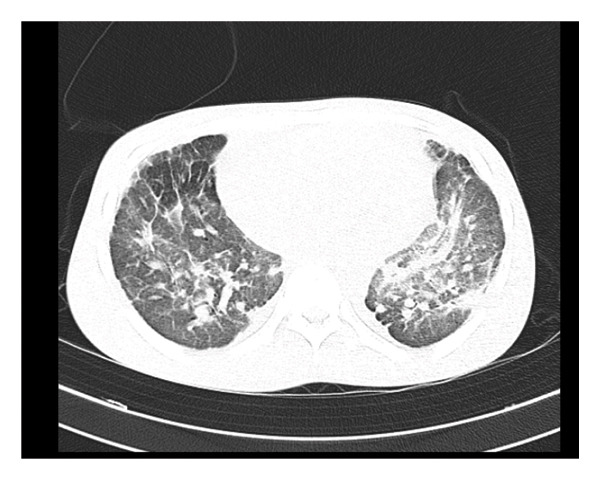
(a)

**Figure:**
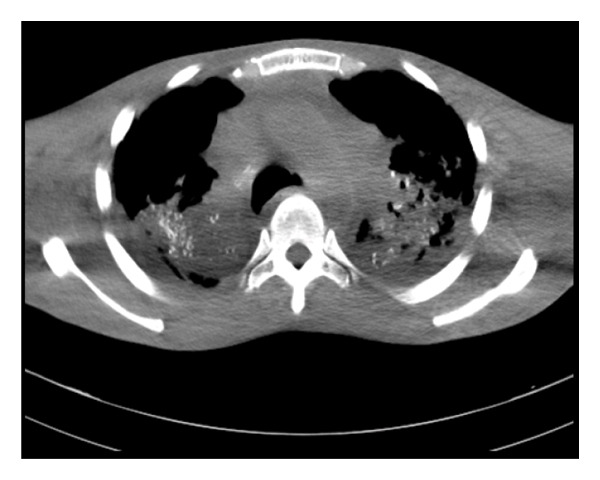
(b)

Based on his history of exposure, radiological evidence for silicosis, and clinical and laboratory manifestations of SSc, the patient was diagnosed with Erasmus syndrome. He was started on immunosuppressive therapy, including oral prednisolone 60 mg daily, which was tapered to 10 mg daily over 4 weeks, and oral mycophenolate mofetil 1 g 12 hourly. He was also prescribed off‐label oral pirfenidone 200 mg 8 hourly and oral omeprazole 20 mg daily. Raynaud’s phenomenon was managed with lifestyle modification and tab nifedipine (extended‐release) 30 mg daily.

Two weeks postadmission, the patient’s oxygen saturation ranged between 88% and 91% on intranasal oxygen at 6 L/min. He was counseled on long‐term oxygen therapy (LTOT) and discharged home with mobile oxygen. There was no significant improvement 1 month after discharge, with an oxygen saturation of 89% on intranasal oxygen and breathlessness at rest. Unfortunately, the patient passed away 8 weeks postdischarge.

## 3. Discussion

Erasmus syndrome, which is characterized by exposure to silica and the subsequent development of SSc, represents a rare yet significant consequence of silica exposure. Silica dust exposure is known to cause silicosis and has been associated with several autoimmune diseases [6, 7]. Silica, the most abundant mineral on earth, exists in two forms, the relatively nontoxic amorphous and the toxic crystalline forms. Industries such as mining (coal, mineral, and metal) and manufacturing (cement, glass, clay, and ceramics) are common sources of crystalline silica exposure [7, 8]. The patient in this report had been an artisanal miner for about 14 years, exposing him to crystalline silica and subsequent silicosis.

Inhalation of crystalline silica causes chronic lung inflammation and fibrosis. Silica particles when ingested by alveolar macrophages lead to cell death and the release of inflammatory mediators such as Interleukin‐1 (IL‐1), IL‐2, and necrosis factor‐α [8]. This triggers T‐helper cells and recruits more macrophages, enhancing the inflammation. The release of cytotoxic substances and proteolytic enzymes by recruited inflammatory cells causes further damage to the lung parenchyma [8]. The ongoing cycle of macrophage activity and cell death drives fibroblast proliferation and collagen deposition, leading to lung fibrosis [8]. The inflammatory mediators released not only perpetuate lung injury but also contribute to systemic autoimmune responses, potentially leading to the development of SSc [9]. The continuous exposure of the patient to silica triggered an autoimmune response that led to SSc.

SSc is a chronic multisystem disease characterized by extensive vascular dysfunction and progressive fibrosis affecting the skin and internal organs [5, 10]. SSc subtypes are categorized by the extent of skin involvement. Limited SSc affects areas distal to the elbows and knees, sparing the face, neck, trunk, and proximal extremities, while diffuse SSc includes the upper arms, thighs, and trunk. Approximately 10% of SSc patients exhibit no skin involvement (SSc sine scleroderma). Some patients may also display features of other autoimmune diseases, such as rheumatoid arthritis and systemic lupus erythematosus (SSc with overlap syndrome) [5, 10].

Features of SSc include skin thickening and hardening with hyperpigmentation and hypopigmentation (“salt and pepper”), dry skin, Raynaud’s phenomenon, telangiectasia, subcutaneous calcinosis, myalgia, arthralgia, arthritis, tendinitis, esophageal dysmotility, pulmonary hypertension, and interstitial lung disease [10–13]. This patient had already developed complications of pulmonary fibrosis and Raynaud’s phenomenon before he was referred to a tertiary facility. There were delays on the part of the primary clinicians to recognize and refer appropriately, as well as to treat the patient for *Mycobacterium TB,* even though the sputum for GeneXpert was negative. We advocate for primary and secondary health clinicians on early referral of patients in situations where they are experiencing diagnostic challenges or do not have the expertise to handle such cases. While clinically and immunologically similar to idiopathic SSc, silica‐induced SSc (SA‐SSc) is more common in males, is associated with severe pulmonary involvement, and has a poorer prognosis. Anti‐Scl‐70 antibodies are the predominant autoantibodies found in SA‐SSc [9, 14–16].

There is no proven cure for Erasmus syndrome, though there has been an improvement in the overall outcome of patients with SSc over the last 4 decades. Regular screening of organs to detect early disease is key in the management [17]. The treatment for SA‐SSc mirrors that of idiopathic SSc, focusing on symptomatic therapy [18]. In patients with diffuse, progressive skin involvement or severe organ involvement, immunosuppressive therapy, such as cyclophosphamide, mycophenolate mofetil, or methotrexate, is recommended. Raynaud’s phenomenon is managed with calcium‐channel blockers, gastroesophageal reflux with proton‐pump inhibitors, and arthralgia or myalgia with short courses of nonsteroidal anti‐inflammatory drugs.

An important complication worth recognition in patients with SSc is scleroderma renal crisis (SRC). This is a rare but life‐threatening complication characterized by malignant hypertension and acute renal failure [19]. It results from endothelial injury caused by a complex interplay of autoimmune, vascular, and molecular mechanisms leading to renal vasculopathy, ischemia, and widespread renin angiotensin aldosterone system activation resulting in malignant hypertension and acute kidney injury [20]. Common triggers include high‐dose glucocorticoids above ≥ 15 mg/day, positive Anti‐RNA polymerase III antibody status, genetic factors such as HLA‐DRB1 ∗ 1407 and HLA‐DRB1 ∗ 1304 susceptibility, volume depletion, and the use of nephrotoxic drugs [20]. The use of corticosteroids in SSc remains controversial as the evidence base is limited, and the risk‐benefit profile is unfavorable, particularly due to the risk of precipitating SRC, especially in patients with early diffuse cutaneous SSc [21, 22]. We recognized that our initiation of 60 mg/day of prednisolone could have induced an SRC. However, this was quickly tapered down over a period of 4 weeks to a dose of 10 mg/day whilst the patient’s blood pressure and renal functions were been monitored.

This study was limited by a lack of pulmonary function test (PFT). Unfortunately, in our setting, spirometry is the only component of PFT that is readily available. We do not have the resources to measure the lung volumes and the diffusion capacity of lungs for carbon monoxide (DLCO). The patient was not stable enough to undergo the spirometry procedure, and hence it was not performed for him. Also, a skin biopsy was not performed for the patient.

In conclusion, this case report highlights the significant health risks associated with silica exposure in artisanal mining, leading to the development of Erasmus syndrome. Preventing silica exposure remains crucial, necessitating stringent workplace safety measures and occupational health practices to protect individuals in high‐risk professions. Clinicians are also advised on early referral of patients to higher centers in situations of diagnostic challenge and lack of expertise.

## Author Contributions

Solomon Gyabaah: conceptualization, data curation, investigation, methodology, project administration, resources, supervision, validation, writing–original draft, and writing–review and editing. Seth Kyei‐Fram: data investigation, methodology, validation, writing–original draft, and writing–review and editing: Kojo Awotwi Hutton Mensah: conceptualization, data curation, investigation, validation, writing–original draft, and writing–review and editing. Osei Yaw Asamoah: writing–original draft, writing–review and editing, investigation, and validation. Clement Ohene Owusu: writing–original draft, writing–review and editing, investigation, and validation. Divine Aseye Yao Amenuke: conceptualization, investigation, supervision, writing–original draft, and writing–review and editing. Yaw Adu‐Boakye: supervision, writing–original draft, and writing–review and editing. Martin Kofi Agyei: supervision, writing–original draft, and writing–review and editing.

## Funding

The authors received no financial support for the research, authorship, and/or publication of this article.

## Consent

Written informed consent was obtained from the patient before his demise to publish this report.

## Conflicts of Interest

The authors declare no conflicts of interest.

## Data Availability

The data that support the findings of this case report are available from the corresponding author upon reasonable request.
